# Rasche und langanhaltende Remission eines therapierefraktären Morbus Hailey‐Hailey durch IL‐13‐Inhibition mit Tralokinumab

**DOI:** 10.1111/ddg.70023

**Published:** 2026-06-04

**Authors:** Oliver Brandt, Stephanie M. Huber, Simon M. Mueller

**Affiliations:** ^1^ Dermatologische Klinik Universitätsspital Basel Basel Schweiz

**Keywords:** Morbus Hailey‐Hailey, Pemphigus chronicus benignus familiaris, Tralokinumab, Dupilumab, Interleukin‐13

Sehr geehrte Herausgeber,

 der Morbus Hailey‐Hailey (HHD; OMIM 169600), auch als Pemphigus chronicus benignus familiaris bezeichnet, ist eine seltene, autosomal‐dominant vererbte, chronisch verlaufende Genodermatose, die sich gewöhnlich im jungen Erwachsenenalter erstmals und mit kompletter Penetranz, aber variabler Ausprägung manifestiert. Betroffen sind die Intertrigines, insbesondere die Axillen‐, Leisten‐ und bei Frauen die Submammärregionen, in denen sich erosive, mazerierte, von Fissuren durchsetzte, erythematöse Plaques entwickeln. Die damit einhergehenden Schmerzen und der häufig vorhandene Foetor führen oft zu sozialer Isolation, was die Lebensqualität der Patienten zusätzlich beeinträchtigt.^1^ Mechanische Irritationen, Schwitzen, UV‐Expositionen sowie mikrobielle und virale Infektionen wirken aggravierend bzw. können einen Schub auslösen.

Ursache der Erkrankung ist eine Mutation in dem für die Kalziumpumpe kodierenden ATP2C1 Gen, wodurch es zu Störungen der intrazellulären Kalzium‐Homöostase und dadurch zu einer beeinträchtigen suprabasalen Keratinozyten‐Adhäsion mit Akantholyse kommt.^2^

Wir berichten über eine 68‐jährige Patientin mit positiver Familienanamnese (Vater sowie Onkel und Großmutter väterlicherseits waren ebenfalls erkrankt), die seit 38 Jahren an einem histologisch gesicherten Morbus Hailey‐Hailey litt, auf zahlreiche erfolglose Therapieversuche nicht oder nur unzureichend angesprochen hat und unter einer Behandlung mit dem Anti‐Interleukin(IL)‐13 Antikörper Tralokinumab erscheinungsfrei wurde.

Die Hautläsionen bestanden zuvor dauerhaft in den Axillen, submammär sowie inguinal und aggravierten während der warmen Jahreszeit regelmäßig durch Schwitzen so stark, dass die Patientin ihre Unterwäsche mit der Innenseite nach außen trug, um zusätzliche mechanische Irritationen durch Nähte zu vermeiden (Abbildung 1a, b). Zudem verzichtete sie während Phasen der Verschlechterung auf sportliche Betätigungen wie Nordic Walking, Schwimmen und Fahrradfahren sowie Aktivitäten, die mit einer Friktion in den betroffenen Körperregionen einhergehen. Rezidivierende Infektionen in den betroffenen Arealen aggravierten den Foetor, weshalb sich die Patientin zunehmend sozial isolierte.

**ABBILDUNG 1:**
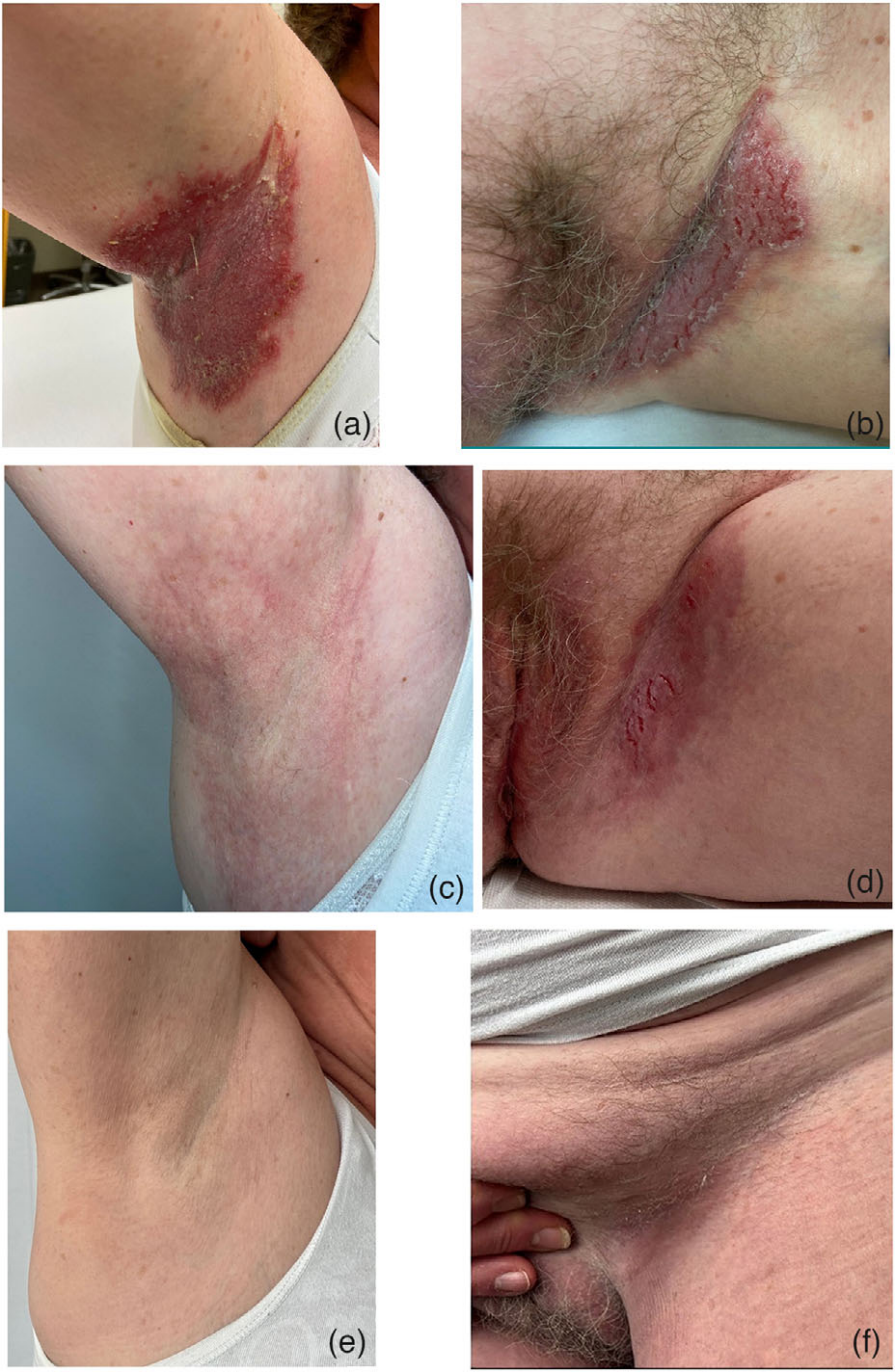
Morbus Hailey‐Hailey Läsionen vor und nach Therapie mit Tralokinumab. (a, b) Erosive, erythematös‐livide, mazerierte, von Fissuren durchsetzte Plaques axillär und inguinal vor Therapiebeginn, (c, d) nach achtwöchiger Behandlung und (e, f) fünf Monate nach der letzten Applikation von Tralokinumab 300 mg.

Behandlungen mit systemischen und topischen Kortikosteroiden, Neotigason, Isotretinoin, Antibiotika und CO_2_‐Lasertherapien jeweils in Kombination mit einmal täglichen Octenidin Umschlägen führten allenfalls zu einer geringen Besserung oder mussten wegen unerwünschter Nebenwirkungen vorzeitig beendet werden.

Nachdem sich der Hautzustand Ende 2023 erneut deutlich verschlechtert hatte und die Patientin aufgrund ausgeprägter Erosionen und starker Schmerzen stationär aufgenommen werden musste, begannen wir eine probatorische off‐label Therapie mit dem IL‐13 Inhibitor Tralokinumab, initial 600 mg, die im Verlauf mit 300 mg alle 14 Tage in Kombination mit den bereits zuvor erfolgten Octenidin Umschlägen einmal täglich fortgeführt wurde.

Bereits wenige Wochen nach der ersten Applikation waren die Läsionen deutlich gebessert und die Patientin war durch die Erkrankung erheblich weniger beeinträchtigt, was sich auch in einem Rückgang des Dermatologischen‐Lebensqualitäts‐Index (DLQI) von initial 26/30 auf 20/30 Punkten widerspiegelte (Abbildung 1c, d). Bis auf eine kurzzeitige Exazerbation nach einem dreiwöchigen Italienurlaub während des Hochsommers, heilten die Hautveränderungen weiter ab, weshalb die Behandlung mit Tralokinumab nach sechswöchiger Erscheinungsfreiheit ebenso wie die antiseptische Lokaltherapie im Oktober 2024 versuchsweise eingestellt wurde.

Mittlerweile ist die Patientin seit elf Monaten symptomfrei, betreibt regelmäßig Nordic Walking, geht Schwimmen und fährt Fahrrad. Eine Behandlung der ehemals betroffenen Körperareale mit freiverkäuflichen Hautpflegecremes erfolgt nur noch nach sportlichen Aktivitäten; der DLQI betrug zuletzt 1/30 Punkten (Abbildung 1e, f).

Die Behandlung des HHD gestaltete sich bis vor kurzem schwierig, signifikante und länger anhaltende Besserungen konnten nur selten erreicht werden.^3^ Während der letzten Jahre wurden mehrere Berichte über die Wirksamkeit des unter anderem für die Behandlung der atopischen Dermatitis (AD) zugelassenen und gegen den IL‐4 Rezeptor gerichteten Antikörpers Dupilumab beim HHD publiziert.^4^, ^5^ Die Autoren beobachteten eine signifikante Besserung oder gar Abheilung der Läsionen binnen weniger Wochen, ohne dass relevante unerwünschte Nebenwirkungen auftraten. Gleiches berichteten Garg et al., die, wie auch wir, ebenfalls einen raschen Wirkungseintritt bei ausgezeichneter Verträglichkeit von Tralokinumab in ihrem kürzlich erschienenen Fallbericht beschreiben.^6^ Die Tatsache, dass beide Antikörper sich als äußerst effektiv in der Behandlung des HHD erwiesen haben, impliziert, dass Typ2‐Entzündungsprozesse maßgeblich für die Pathophysiologie der Erkrankung sind. Allerdings sind die genauen Wirkmechanismen bislang nicht bekannt.

In den letzten Jahren wurde die prominente Rolle deutlich, die IL‐13 nicht nur bei der AD, sondern auch bei anderen chronischen Entzündungen in der Haut spielt.^7^ Das Zytokin wird auch nach Barriereschädigung durch Type2‐Innate Lymphoid Cells (ILC2) und vor allem von den durch diese via Keratinozyten‐Alarmine rekrutierten Th2‐Zellen exprimiert. Es ist somit denkbar, dass die in Keratinozyten infolge der gestörten Ca2+ Homöostase induzierte oxidative Stressreaktion eine mit einer Barriereschädigung einhergehende Entzündungsreaktion auslöst, die dann durch IL‐13 aggraviert und unterhalten wird.^8^ Zudem konnte in in‐vitro Experimenten gezeigt werden, dass IL‐4 und IL‐13, jedes für sich, als auch in Kombination, die Ca^2+^‐Mobilisation in Keratinozyten beeinflussen und deren Differenzierung inhibieren,^9^ sowie die Expression von Struktur‐ und Adhäsionsmolekülen, wie beispielsweise Desmoglein1 und Desmocollin1, vermindern. Letzteres könnte Ursache für die beim HHD regelmäßig nachweisbare Akantholyse sein.^10^ Eine Hemmung von IL‐13 durch Tralokinumab oder Dupilumab könnte somit deren ausgezeichnete Wirksamkeit beim HHD erklären.

Unsere Beobachtung, dass die selektive Inhibition von IL‐13 ausreichend für eine effektive Behandlung des HHD und sogar krankheitsmodifizierend sein kann, deutet darauf hin, dass dieses Zytokin, ebenso wie bei der atopischen Dermatitis, maßgeblich an der Unterhaltung des chronischen Entzündungsgeschehens beteiligt ist und somit eine bedeutsame Rolle in der Pathogenese des HHD spielt. Studien werden zeigen müssen, ob Medikamente, die die durch IL‐13 vermittelte Entzündungsreaktion hemmen, zu einem Standard in der Behandlung des HHD werden können.

## INTERESSENKONFLIKT

Oliver Brandt hat von folgenden Pharmaunternehmen Zuschüsse für Kongressbesuche erhalten oder war für sie als Berater tätig: Abbvie, Bayer, Beiersdorf, Jansen/ Johnson & Johnson, Leo Pharma, Novartis, Pfizer, Sanofi, UCB. Stephanie M. Huber hat Zuschüsse für Kongressbesuche von Merz Pharma Schweiz und Stallergenes erhalten. Simon Müller erklärt, dass er in den letzten drei Jahren als Berater und/oder Referent Honorare und/oder Zuschüsse erhalten hat und/ oder als Prüfer für klinische Studien folgender Unternehmen tätig war: Sanofi‐Aventis AG, Galderma SA, Janssen‐Cilag AG, LEO Pharmaceutical Products Sarath, medtis GmbH, Amgen, Incyte, La Fonderie SAS, Novartis Pharma AG.

Open access publishing facilitated by Universitat Basel, as part of the Wiley ‐ Universitat Basel agreement via the Consortium Of Swiss Academic Libraries.
